# Response To: Investigating sources of inaccuracy in wearable optical heart rate sensors

**DOI:** 10.1038/s41746-021-00408-5

**Published:** 2021-02-26

**Authors:** Peter J. Colvonen

**Affiliations:** 1 VA San Diego Healthcare System, San Diego, CA USA; 2 University of California, San Diego, Department of Psychiatry, San Diego, CA USA; 3 VA Center of Excellence for Stress and Mental Health, San Diego, CA USA; 4 National Center for PTSD, White River Junction, VT USA

**Keywords:** Medical research, Health care

**Arising From** Bent et al. *npj Digital Medicine* 10.1038/s41746-020-0226-6 (2020)

Recently, Bent and colleagues (2020) published a timely and well-written paper examining the role of skin tone on inaccurate readings in consumer and medical grade wearables (Empatica E4 + ; Apple Watch 4; Fitbit Charge 2; Garmin Vivosmart 3; Xiaomi Miband; Biovotion Everion)^1^. They found no significant difference in accuracy across skin tones, but did find differences by devices in response to changes in activity. This finding is in contrast to previously reported studies finding wearables using green light technology had larger errors rates in tracking heart rate and energy expenditure for individuals with darker skin tones^2^, especially if exercising^3^. So while Bent and colleagues’ paper is a model in reporting in many ways, due to the incredibly important nature of this topic, it is crucial to appraise their paper to advance scientific discourse and highlight several recommendations for future researchers. Specifically, I believe their findings may be misleading due to their small sample size, which may miss important interaction effects of confounding variables and skin tone, and their use of the Fitzpatrick Skin Type Scale, which has a substantial literature of racial biases^4–6^, weak correlation with skin color, and large within-group variations of skin tone^4,7,8^. As such, I am concerned their findings on skin tone are not accurate and will be used to limit or misrepresent future research on inaccuracies of skin tone in wearable devices.

It is estimated that by the year 2021 there will be 121 million Americans using wearable devices^9^. Wearables promise a myriad of health-related information, including low heart rate alerts, a personal electrocardiogram (ECG) monitor for detecting arrhythmia, sleep tracking (e.g., sleep architecture), and pulse pressure designed to promote healthy living and alert high-risk consumers based on real-time data. Their relative low cost, the collection of longitudinal data, and ability to display/transmit information suggests a host of benefits if used in clinical practice and to advance remote research. The concern is, as highlighted by Ruth Hailu in a media article in 2019^10^, due to technological limitations of Photoplethysmography (PPG) green light signaling, these health constructs may not be as accurate for a population of people with darker skin tones^2,3^. While newer versions of wearables (e.g., Apple Watch 6) have added pulse oximeters, there is evidence that pulse oximeters also have increased error rates based on skin tone^11,12^. Further, these devices are now transitioning from consumer goods into health-related research and their internal algorithms are becoming FDA approved. This is concerning because if there are significant errors by skin tone that are not specifically examined it can limit accurate health-related information for individuals with darker skin tones, further exacerbating already existing structural health disparities^13^. Our challenge in the scientific community is to examine and accurately report the validation of PPG technology for individuals with dark skin.

Despite thousands of published articles on wearables (e.g., Fitbit alone has approximately 476 published studies and 449 studies registered on ClinicalTrials.gov^1^ and the Apple Watch Heart Study recruited 419,927 people to track irregular heart rhythms)^14^ there are only a small handful of studies that examine skin tone and accuracy rates directly^1–3,11^. A lack of accurate information about error in diverse skin tones may cause unintended consequences by limiting access to accurate health information based on skin tone and reinforcing existing healthcare disparities. As such, the Bent and colleagues’ design, methods, and reporting holds lessons that should be modeled for future studies. For example, they run a sophisticated study using the current gold-standard measures and have a strong reference group (in this case they used medical grade ECG as their comparison). Further, they present their results with all confidence intervals, error rates (in their case they actually provide different two forms of errors rate: mean directional error and the mean absolute error), and missing data for each device, skin tone group, and activity. However, there are two aspects that need to be improved upon for future research: skin tone classification and sample size.

As I noted in Colvonen and colleagues (2020), a major confounding factor in accurately understanding the limitations of wearables on skin tone is the current gold standard of measuring skin tone: the Fitzpatrick Skin Type Scale (FST)^15^. Developed in 1975 by individuals with white skin for individuals with white skin^6^, the FST is a subjective scale that classifies six skin type categories according to the amount of skin pigmentation and skin’s reaction to sun exposure. There is a substantive literature examining the racial biases and limitations of the FST^4–6^. Ware and colleagues (2020) point out that the FST was originally used to assess the propensity for skin to burn, and only later became a means of describing skin tone. This is consistent with the findings that phototype designation of six categories has been shown to have only a weak correlation with skin color that results in large within-group variance of skin tone^4,7,8^. I hypothesize that wearables may not work well with darker skin tones, or a combination of darker skin tones and confounding variables, that are a subset of the FST Type 6 group. Due to the large within-group variation of FST skin tone classifications, errors in wearables in darker skin tone subsets are likely to be missed.

Further, the FST has been shown to be inaccurate and biased based on the administrator^16^. For example, Fider and Komarova^16^ found that men and women classify color grouping markedly different. As such, the use of the subjective FST may not accurately classify skin tone based on the administrator. While there are other skin tone scales that offer more skin tone categories (e.g., Taylor Pigmentation Scale), this does not fix the problem of the subjective nature of classifications. The best solution is to stop the use of subjective skin tone scales altogether. I recommend replacing it with objective reflectance spectrometry which accurately identifies skin color/tone using multiple color wavelengths for classification^17^, and should be the new gold standard for all studies examining wearables. Spectrocolorimetry generally uses multiple variables for categorizing skin tone. The most common variables are lightness/brightness (a gray scale from pure white to pure black), red/green value, and a blue/yellow value that more accurately represents empirical values of color tones^18^. Some colorimeters are able to not only assess skin color’s full spectral characteristics but also cutaneous (skin/fat layers) physiology (see Ly and colleagues^19^ guide to research techniques for colorimeters)^19^.

While Bent and colleagues ran a power analysis to address sample size, I fear that their conclusions may be misleading as too few people with the darkest skin tones were included (*n* = 9 in FST Type 6). There are several factors that influence PPG accuracy that may cause an interaction with skin tone, including the presence of arm hair, sweat, ambient temperature, level of activity, thickness of skin epidermis^20^, and body mass^21^. Taken together with the within-group variance of skin tone in the FST and human error for classifying skin tone categories, it is not surprising the Bent and colleagues did not find differences in error rates by skin tone. I recommend the future research of skin tone accuracy and wearables to increase their sample size to account for possible interactions with skin tone, and to allow a large enough sample of darker skin tones to limit false negatives.

Our challenge as scientists is to fully and accurately represent the possible limitations of PPG technology for individuals with dark skin to limit any unintentional contribute to health disparities. Taken together, it is vital that we work together to raise the bar in running high quality studies and accurately reporting objective findings to ensure that digital health solutions do not reinforce existing disparities in care and access as wearables are increasingly used in research and clinical practice. This should include: 1) decreasing use of the subjective skin tone measures and increasing reporting of objective, non-offensive, standards of skin tone; 2) increasing sample sizes to allow for interaction effects on skin tone; 3) directly working with wearables companies to improve upon their effectiveness and consumer reach to support people of color; 4) holding the research community accountable for addressing and reporting bias; and 5) making sure that people of varying skin tones are included in validation and effectiveness research.
